# Inbreeding Coefficient and Distance in MHC Genes of Parents as Predictors of Reproductive Success in Domestic Cat

**DOI:** 10.3390/ani12020165

**Published:** 2022-01-11

**Authors:** Mariya N. Erofeeva, Galina S. Alekseeva, Mariya D. Kim, Pavel A. Sorokin, Sergey V. Naidenko

**Affiliations:** 1 A.N. Severtsov Institute of Ecology and Evolution, Russian Academy of Sciences, Leninsky pr. 33, 119071 Moscow, Russia; gal.ser.alekseeva@gmail.com (G.S.A.); sorokin-p@yandex.ru (P.A.S.); snaidenko@mail.ru (S.V.N.); 2 Department of Zoology, Institute of Zootechnics and Biology, Moscow Timiryazev Agricultural Academy, Russian State Agrarian University, Timiryazevskaya Str. 49, 127550 Moscow, Russia; marykim1069@yandex.ru

**Keywords:** inbreeding, MHC, reproductive success, survival rate, body mass, domestic cat

## Abstract

**Simple Summary:**

Inbreeding and low diversity in MHC (major histocompatibility complex) genes can have a significant impact on the survival and quality of offspring in mammals. At the same time, a decrease in genetic diversity can be disastrous for animals at individual and species level. For felines, studies of the effects of inbreeding and low variety in MHC genes are conducted on populations with a low number of animals, where there is a high probability of a shortage of available partners, and, accordingly, their choice. The use of model species, especially domestic cats, allows us to identify the main consequences of inbreeding and the lack of a choice of partners for future offspring. The survival of offspring in a domestic cat is primarily affected by the degree of similarity/difference in the genes of the parents’ MHC. Parents with the maximum distance in MHC genes have a larger proportion of surviving kittens, and this effect is most pronounced immediately after birth. In parents with the minimum distance in MHC genes, a significant percentage of kittens are either stillborn or die on the first day after birth. However, inbreeding and the similarity of parents in MHC genes in domestic cats did not affect the body mass of kittens.

**Abstract:**

Inbreeding and low diversity in MHC genes are considered to have a negative effect on reproductive success in animals. This study presents an analysis of the number and body mass of offspring in domestic cat, depending on the inbreeding coefficient and the degree of similarity in MHC genes of class I and II in parents. Inbred partners had a lower number of live kittens at birth than outbred ones. At the same time, the inbreeding coefficient did not affect the litter size and the number of offspring who survived until the period of transition to solid food. The most significant predictor for the number of surviving offspring was the degree of parental similarity in MHC genes: the parents with the maximum distance in MHC genes had more survived kittens. Moreover, this effect was most pronounced immediately after birth. A significant percentage of kittens from parents with a minimum distance in MHC genes were either stillborn or died on the first day after birth. By the age of transition to solid food, this effect is no longer so pronounced. Furthermore, neither the inbreeding coefficient nor the distance in MHC genes of parents had any effect on the body mass of kittens.

## 1. Introduction

The high density of free-ranging cats can be a serious problem to safeguarding their well-being. A high number of animals and the promiscuous mating system contributes to their vulnerability to various diseases and strong parasite pressure [1,2]. At the same time, low genetic diversity can lead to the instability of animals in the population to diseases [3,4,5]. A decline in genetic diversity can be detrimental to animals, both at the individual and species level. Inbreeding can have a significant effect on the survival rate and quality of offspring, and, accordingly, affect the survival of the species [6]. The influence of inbreeding on reproductive success has been shown in many animals [7,8,9,10]. It has been shown that inbred parents have a smaller number of offspring [8,9], they are of inferior quality [7,11,12] and their survival rate is lower, in contrast to offspring born from outbred parents [9,11,12,13]. Descendants from inbred parents themselves also have lower reproductive success in the future [7,12,14]. Cases of the existence of groups with low genetic diversity have been described in felines (cheetah [15], Amur leopard [16], Amur tiger [17,18]). However, for the Eurasian lynx in captivity, it was shown that the negative effect of inbreeding on the body mass of cubs leveled out by the age of three months [19].

High polymorphism of the MHC (major histocompatibility complex) genes ensures the body’s resistance to many pathogens, and thus increases its fitness [20,21,22]. It is “beneficial” for parents to have offspring from a partner that is more distant in the MHC genes, ensuring maximum heterozygosity for the offspring, which increases its fitness. It is believed that choosing a partner with MHC genes the most different from theirs allows females to produce the most viable offspring (with an accelerated rate of development and a strong immune system). The influence of MHC genes on mate choice has been confirmed in a few invertebrates and vertebrates, including mammals [23,24,25,26,27,28,29,30,31] and humans [29,32,33]. However, there is currently insufficient information on the consequences of such a choice for several species (or systematic groups), including felines. For some representatives of the feline family, a decrease in the body mass of cubs produced from inbreeding has been described [11,19]. At the same time, there is no information on the influence of the choice of MHC genes partners on the quality and survival of the offspring.

Studies of the impact of genetic diversity on breeding success tend to focus on rare and endangered animal species. To date, the number and density of populations of most felines in nature decreases. In such a situation, there may be limitations on the number of available partners, and, accordingly, on their choice. Understanding the possible negative consequences for offspring in such populations can be important for the conservation of rare and endangered mammalian species. However, given the lack of knowledge on this issue, it seems important to us, first of all, to carry out such studies on numerous and widespread species. Understanding the level at which the negative effects of reduced genetic diversity begin to manifest will help to better predict the impact of inbreeding and/or low variety in MHC genes in populations of rare and endangered animals on the likelihood of their extinction/conservation.

The domestic cat (*Felis catus*) is the most widespread species of felines and forms free-living populations of feral individuals on all continents (except Antarctica) and several oceanic islands. It is the most abundant feline species [34,35]. This species is characterized by high genetic diversity [36,37] and is very dependent on humans, or rather on the density of the population and the degree of development of the area by humans. As a rule, in cities, the population density of free-living domestic cats is much higher than in rural areas (2–3 thousand individuals/km^2^ versus 0.25 individuals/km^2^, respectively) [38,39]. In turn, such variety in population density leads to significant differences in the lifestyle of free-living domestic cats. At a low density, a solitary lifestyle and a monogamous mating system are more typical for the domestic cat [40,41,42,43], while at a high density, a social lifestyle and promiscuity are typical [42,43,44,45]. Given the large number and the lifestyle of this predator, the risks of negative impact from low genetic diversity in natural populations should be rather small. However, testing this hypothesis in populations of free-ranging cats, especially in populations of high density, is difficult. A female can mate with up to nine males during the mating season [44]. In such a situation, it can be complicated to determine not only the paternity of kittens but also to assess the genetic diversity of potential parents. At the same time, even with constant monitoring of the group of free-ranging cats, estimation of the number of stillborn kittens and kittens that die during the denning period is almost impossible. A significant factor influencing the survival rate of the offspring is the food resource, which in natural conditions can be irregular and poorly monitored. The aim of our study was to evaluate, based on a long-term array of data on the reproduction of domestic cats at the biological station “Tchernogolovka”, the reproductive success, growth rate (body mass) and survival of kittens depending on the parental inbreeding coefficient and the degree of similarity in MHC genes in the domestic cat. We assume that even in a domestic cat, as a species with high genetic diversity, a high degree of kinship and a smaller distance in the MHC genes leads to (1) a decrease in the number of born and surviving offspring; (2) a decrease in the growth rate (body mass) of the offspring.

## 2. Materials and Methods

This study was conducted at the Joint Usage Center “Live collection of wild species of mammals” at A.N. Severtsov Institute of Ecology and Evolution (the biological station “Tchernogolovka”), Russia. This station is located 50 km northeast of Moscow (56°00′ nl, 38°22′ el). The Institute keeps the breeding colony of domestic cats for behavioral and physiological research [46,47,48,49]. The founding animals were obtained from the feral cats’ population in Moscow in 2008, approximately. The founders of the population were 19 (10 females, 9 males) free-ranging domestic cats brought from feral populations in different districts of Moscow. All but two of the animals (1 male and 1 female, siblings) were unrelated. By 2021, the number of cats in the colony was 66 individuals (22 males, 44 females). Of these 66 animals, 10 (7 females and 3 males) were brought from natural populations, the rest were born at the biological station “Tchernogolovka” during previous studies. The husbandry conditions were described in detail earlier [46,47,48,49]. Domestic cats, except for nursing females with kittens, were housed individually in cages (2 × 1(1.5) × 2 m size) all year. Females and males were housed together only during the mating period. The daily food ration consisted of approximately 200 g chicken meat supplemented with vitamins and minerals, and water was provided ad libitum.

Studies of reproductive strategies have been conducted on the studied population for more than 10 years. Mating pairs were selected by researchers. All matings were also carried out under the observation of researchers. Therefore, we have always known the potential fathers, the date of mating, the date of birth and the number of offspring born. The matings were carried out only during the estrus period in the female, which was determined by her behavior [47]. The animals were kept in outdoor enclosures all year round. The mating period was limited by climatic conditions: kittens were born either in late spring or summer. Despite the fact that the domestic cat is capable of producing up to three litters per year, in our colony we bred each female only once a year. This was necessary in order to exclude too high a “reproductive investment” that could affect the number and quality of future offspring [50,51].

All studied kittens were born at the biological station “Tchernogolovka” between 2011 and 2021. We checked the number and condition of kittens on the first day after the birth and every two weeks after it. The kittens were weighed with the hand-scale RST (Lund, Sweden) with precision to 10 g. To analyze the influence of the IC (inbreeding coefficient) and distance in MHC genes on reproductive success, we estimated the litter size at birth and the number of alive kittens and their body mass at two age periods. The first period was the first day after birth, when kittens cannot survive without maternal care: they are helpless and fed only on mother’s milk. The inspection of the litter always took place on the first day of the kittens’ life. The second period was the age of the change in diet and the transition to self-feeding with solid food (60 days). At this age, kittens were no longer dependent on their mother’s milk and the basis of their nutrition was solid food, the maternal effect on the development of kittens during this period was already minimal. The inspection of the litter took place on day 58–62 of the kittens’ life.

To analyze the influence of the IC (inbreeding coefficient) and distance in MHC genes on reproductive success, we used only those litters for which the condition of kittens was known in both age periods and the paternity was established clearly. In total, we analyzed 62 litters (223 kittens) from 30 females, of these, 13 litters were obtained from previously non-breeding females.

The cat colony at “Tchernogolovka” station has been reproducing since 2008, and the pedigree of all animals is known. Thirty females and 17 males were the parents of the kittens in this study. The inbreeding coefficient (IC) or coefficient of relatedness was calculated for each pair of parents [52,53,54]. We estimated this IC as the percentage of common genes of two animals (mother–offspring and siblings have 0.5; half-siblings and grandparents–grandchildren 0.25, etc.). We considered the litters/kittens that were mothered by the parents with IC = 0 as outbred, and we considered all others as inbred litters. The IC for the parents of inbred litters varied from 0.12 to 0.5.

Since 2015, we have had the opportunity to genotype our cat population by the genes of the MHC class I and II. MHC class I and II gene distances were determined only for 29 of the 47 cats studied (20 females, 9 males). Thus, we had information about the distance of the MHC class I and II genes between parents for 38 litters (131 kittens). MHC class I and II gene distances ranged from 0 to 1, where 0 is the minimum distance and 1 is the maximum. 

All genetic studies were carried out in the Center for collective use „Instrumental methods in ecology” of A.N. Severtsov Institute of Ecology and Evolution, Russian Academy of Sciences.

For molecular genetic testing, blood was obtained from annual veterinary examinations of animals. Up to 1 mL of blood was taken from the external inguinal vein into a test tube with K3-EDTA buffer and frozen at −20 °C until studies. To determine the distance according to MHC class I and II, DNA isolation from blood samples from 29 cats (20 females, 9 males) was performed with a DNeasy Blood and Tissue Kit (Qiagen, Düsseldorf, Germany). To assess the genetic distances between animals, we used microsatellite loci linked to the MHC I and MHC II genes. We performed polymerase chain reaction (PCR) with Bio-Rad T100 Thermal Cycler (Bio-Rad, Hercules, CA, USA) in 10-μL volumes with final concentration: 0.05 mM of each deoxyribonucleotide triphosphate (dNTP), 2.5 millimoles of MgCl_2_, 0.5 picomoles of the forward and reverse primers, 1 unit of Hot Start Taq DNA polymerase (SibEnzyme, Ltd., Novosibirsk, Russia), 1 × PCR buffer and 1.0 μL of DNA extract. We used 6 pairs of primers with fluorescent dyes for microsatellite loci: MHCI-A rox, MHCI-B fam, MHCI-C tamra, MHCI-D tamra, and MHCII-A fam, MHCII-B r6g [55]. Parameters for the PCR were: 1 cycle of 95 °C for 3 min, 33 cycles of 94 °C for 30 s, 54–61 °C for 30 s, 72 °C for 30 s, and 1 cycle of 72 °C for 30 min. The lengths of microsatellite fragments were determined on an ABI 3500 genetic analyzer (Applied Biosystems, Waltham, MA, USA) in a POP7 polymer with a Liz 500 size standard and GeneMapper v 4.0 software (Applied Biosystems, Waltham, MA, USA). Genetic distances (DA distance) were calculated in the POPTREE2 program using the formula [56,57]. The distance for the MHC class I genes between the parents ranged from 0.27 to 1, where 0.27 was the minimum distance and 1 was the maximum. The distance for the MHC class II genes between the parents varied from 0 to 1, where 0 was the minimum distance and 1 was the maximum. 

In the analysis, we used litters obtained from different mating systems: monogamy and promiscuity. For litters obtained from a promiscuous breeding system, it was necessary to determine the paternity of the kittens. Paternity was determined for 27 litters (97 kittens) using microsatellite analysis. DNA isolation from blood samples from cats was performed with a DNeasy Blood and Tissue Kit (Qiagen, Düsseldorf, Germany). We performed polymerase chain reaction (PCR) with Bio-Rad T100 Thermal Cycler (Bio-Rad, Hercules, CA, USA) in 10-μL volumes with final concentration: 0.05 mM of each dNTP, 2.5 mM of MgCl2, 0.5 picomoles of the forward and reverse primers, one unit of Hot Start Taq DNA polymerase (SibEnzyme, Ltd., Novosibirsk, Russia), 1 × PCR buffer and 1.0 μL of DNA extract. We used 11 pairs of primers with fluorescent dyes for microsatellite loci and 1 pair of primers for sex chromosome: Fca 733-fam, Fca 723-fam, Fca 731-fam, Sry-r6g, Fca 441-r6g, Fca 736-r6g, F 124-r6g, F 53-rox, Fca 749-rox, Fca 742-tamra, F 85-tamra, Fca 740-tamra [58]. Parameters for the PCR were: 1 cycle of 95 °C for 1 min, 35 cycles of 94 °C for 1 min, 59 °C for 1 min, 72 °C for 1 min, and 1 cycle of 72 °C for 45 min. The lengths of microsatellite fragments were determined on an ABI 3500 genetic analyzer (Applied Biosystems, Waltham, MA, USA) in a POP7 polymer with a Liz 500 size standard and GeneMapper v 4.0 software (Applied Biosystems, Waltham, MA, USA). Direct paternity was determined manually by checking the absence or presence of paternal alleles for each of the 11 microsatellite loci in offspring. Paternity was considered confirmed if at each locus in the offspring, in addition to one of the two maternal alleles, one of the two alleles of the potential father was present.

For statistical analysis of the data obtained, we used Statistica 10.0 and the R 4.1.0 environment [59]. We used linear mixed-effect models (LMM) to analyze the influence of the IC and age period on litter size, the number of surviving offspring at age 0 (some of the kittens could be stillborn) and 60 days, and the body mass of kittens (at the age of 0 and 60 days) using the nlme4 library in the R environment package [60]. We compared candidate models with all predictor combinations including factor interactions using model selection based on the Akaike Information Criterion (AICc) in the MuMIn library [61,62]. The mating system was also taken into account in the model. AICc weights were calculated, and the coefficient estimates with their errors were averaged for models with ΔAICc < 2 [63]. The identity (ID) of the litter was included in all models as a random factor. Post-hoc comparisons were made with the Tukey HSD test using the emmeans package in R (Supplementary Materials: File S1: IC) [64]. The effect of distance by MHC genes and age period on litter size, number of surviving offspring and kittens’ body mass was also analyzed using LMM using the nlme4 library in the R environment package [60]. We also compared candidate models with all predictor combinations including factor interactions using the Akaike Information Criterion (AICc) model selection in the MuMIn library [61,62]. At the same time, the model took into account the parental IC and the mating system. AICc weights were calculated, and coefficient estimates with their errors were averaged for models with ΔAICc < 2 [63]. The identity (ID) of the litter was included in all models as a random factor. Post-hoc comparisons were made with the Tukey HSD test using the emmeans package in R (Supplementary Materials: File S2: MHC) [64].

## 3. Results

To analyze the influence of the inbreeding coefficient on the number and quality of offspring, we analyzed all litters of kittens (62 litters, 223 kittens) born at the biological station “Tchernogolovka” from parents with a known pedigree. The average litter size was 3.6 ± 0.2 kittens per female. By the age of transition to solid food (60 days), the average litter size (surviving offspring) was 2.4 ± 0.2 kittens per female (the overall mortality rate was 33%). The most appropriate model with the lowest AICc for surviving offspring included IC, age period, and litter size as significant predictors. The mating system was not a significant predictor and was not included in the model. The litter size at birth was significant for the number of surviving offspring (B = 0.77 ± 0.09, z = 8.12, *p* < 0.0001, Table 1).

By the period of transition to solid food, the number of kittens in large litters (more than 5) decreased by 1.8 times (mortality 44%), and in small litters (1,2 kittens) by 1.3 times (mortality 17%) (Tukey post-hoc test, t = 4.7, *p* < 0.0001) (Table 2). The IC did not affect either the litter size at birth or the number of live offspring at that moment (Table 1). Only if we exclude from the model the most significant predictor “litter size at birth”, does the correlation between the IC and the number of surviving offspring become apparent (B = −2.2 ± 1.05, z = 2.07, *p* = 0.04). From unrelated parents, the average number of live kittens at birth was 3.13 ± 0.23 kittens per female; for parents with an IC of 0.5 this was 2.11 ± 0.3 kittens per female (Table 2). At the same time, the average litter size was not related to the IC.

The most efficient model is the one with the lowest AICc for the average body mass of a kitten in the litter, with the litter size and number of live kittens as significant predictors. We considered each age period separately to exclude the influence of age. We did not find any significant effect of these predictors on the body mass of kittens at birth (Table 1). However, at the age of transition to solid food (60 days), the body mass of kittens depended on the number of surviving offspring (B = −0.03 ± 0.02, z = 2.05, *p* = 0.04). The body mass of kittens was higher in litters with fewer surviving offspring.

For some of the kittens we studied, we had information about the distance by genes MHC class I and II between parents (38 litters, 131 kittens). The average litter size from parents with a known distance by genes MHC class I and II was 3.4 ± 0.2 kittens per female. By the age of transition to solid food, the average litter size (surviving offspring) was 2.3 ± 0.2 kittens per female (mortality rate 32%). The most appropriate model with the lowest AICc for surviving offspring included MHC I and MHC II distance, age period and litter size as significant predictors. The mating system and parental IC were not significant predictors and were not included in the model. Litter size at birth was most significant for the number of surviving offspring (B = 0.74 ± 0.12, z = 6.13, *p* < 0.0001, Table 3).

**Figure 1:**
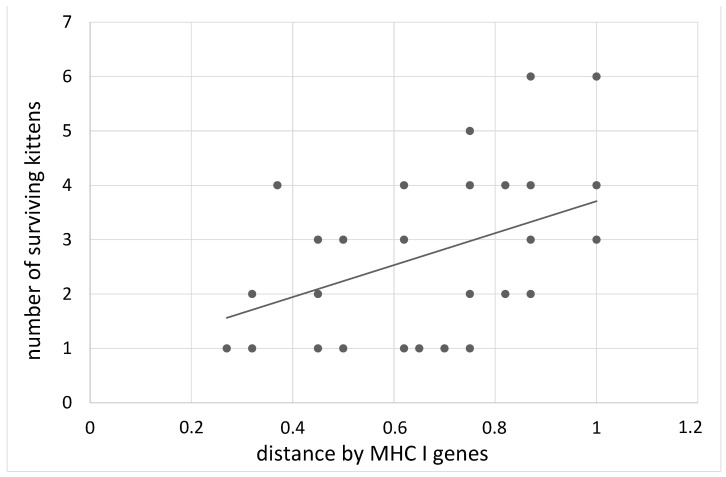
The dependence of the number of kittens born alive on the distance of the MHC I genes of the parents.

By the period of transition to solid food, the number of kittens in large litters (more than 5) decreased by 1.7 times (mortality 41%), and in small litters (1,2 kittens) by 1.2 times (mortality 17%) (Tukey post-hoc test, t = 3.7, *p* < 0.001). In litters born to parents with the maximum distance in the MHC genes of I and II classes, more kittens survived (B = 2.62 ± 0.99, z = 2.55, *p* = 0.01; B = −1.41 ± 0.7, z = 1.94, *p* = 0.05, Table 3). At the same time, this effect was most significant in relation to class I of MHC. In parents with the maximum distance in class I MHC genes, 91.8% of kittens were alive on the first day after birth, while in parents with a minimum distance this index was 71.4% (B = 2.55 ± 0.82, z = 3.0, *p* = 0.003, Figure 1).

**Figure 2:**
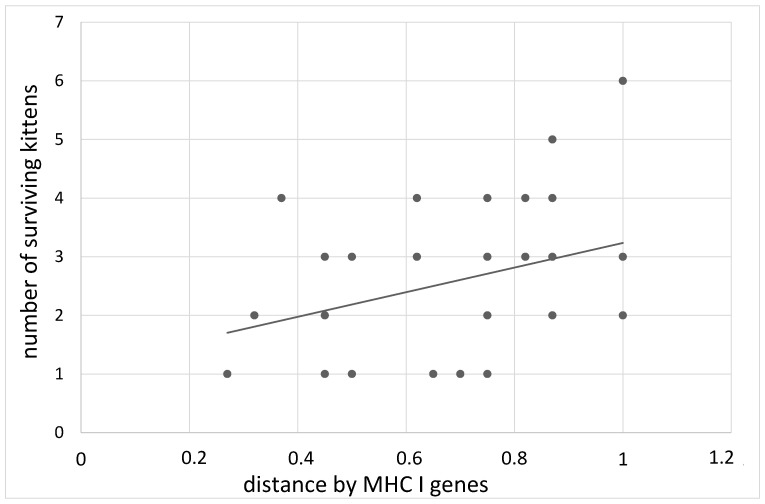
The dependence on the number of living kittens at the 60th day of age on the distance of the MHC I genes of the parents.

By the age of transition to solid food, 84.9% of kittens survived from parents with the maximum distance in the MHC class I genes, and 69.5% survived from parents with the minimum distance (B = 2.52 ± 1.27, z = 1.92, *p* = 0.05, Figure 2). The distance effect for class II MHC genes manifested itself somewhat differently. At birth, the largest number of live kittens observed in parents with a distance of 0.6–0.8 was 83.6%, in other cases, the percentage of surviving kittens was about 75% (B = −1.23 ± 0.53, z = 2.23, *p* = 0.02). By the age of transition to solid food, this effect disappears (B = −1.55 ± 0.99, z = 1.52, *p* = 0.13).

The most efficient model is the one with the lowest AICc for the average body mass of a kitten in a litter, in which only the litter size and number of live kittens are as significant predictors. We considered each age period separately to exclude the influence of age. We did not find any significant effect of these predictors on the body mass of kittens either at birth or during the transition to solid food.

## 4. Discussion

The most important indicator of the adaptability of organisms to environmental conditions is their reproductive success (the number of viable and fertile offspring) [65]. To maximize reproductive success, females of many mammalian species demonstrate preference for a mating partner, which ensures the genetic advantages of offspring [66,67,68]. The choice of a mating partner can have a significant impact on the fertility and reproductive success of animals [47,69,70,71,72,73]. In the domestic cat, as a rule, males are larger than females, which makes it difficult for a female to choose a partner [47,49]. Nevertheless, in the domestic cat, females can demonstrate a preference for a mate [47,74], while females can influence the competition of male sperm, and assessment of the quality of partners and their genetic compatibility can occur after mating in the female reproductive organs (“hidden female choice”) [75]. However, to what extent is the effect of such a choice demonstrated in the number and survival of offspring in domestic cats? In this study, we tried to trace the correlation between reproductive success (the number of offspring, their survival rate and their body conditions) and the degree of relationship and distance in the MHC genes of the parents.

It is believed that mating with related partners leads to negative consequences for their offspring. For many animal species, a high mortality rate has been shown for the young born to inbred parents compared to the young from outbred parents [9,11,76,77]. For some species, this figure can be very high. For example, the survival rate of red deer (*Cervus elaphus*) calves born to inbred parents is 77% lower in the first year of life compared to the offspring from outbred parents [13]. Our study also noted the influence of the degree of relationship of partners on breeding success in the domestic cat. Inbred parents had fewer live kittens at birth than outbred ones. At the same time, the IC did not affect the litter size and the number of offspring that survived until the period of transition to solid food (60 days). The body mass of kittens also did not depend on the parental IC, although such a relationship was shown for many animals, including felines. A decrease in the survival rate and body mass of cubs during the reproduction of parents with a high IC has been described [11] for the Sumatran tiger (*Panthera tigris sumatrae*). In the Eurasian lynx (*Lynx lynx*), cubs born to inbred parents weigh less than those born to outbred parents [19]. For canines in captivity, similar effects of inbreeding were observed [7,12]. Moreover, in wolves (*Canis lupus*) during inbred reproduction, not only a decrease in the mass of offspring was noted but also a lower survival rate of offspring [12]. Moreover, with inbred breeding, they produced blind pups [7,12]. In domestic cats, the effect of inbreeding was not so significant and was manifested only in the number of live kittens at birth. In general, not all animals [10,78], including felines, demonstrate a negative effect of inbreeding on reproductive success and offspring quality. For example, in the cheetah (*Acinonyx jubatus*), only a slight decrease in the body mass of cubs was shown with inbred breeding [79].

However, the consequences of inbreeding can be prolonged. Offspring from inbred parents may have lower reproductive success in the future [7,12,14]. For example, for the Mexican gray wolf (*Canis lupus baileyi*), a decrease in the quality of sperm in inbred males and, as a consequence, a decrease in their reproductive success in the future has been described [7]. This is also true for domestic cats. Inbreeding leads to a decrease in the quality of sperm in males, the proportion of abnormal spermatozoa increases [46,80], whereas a high proportion of abnormal spermatozoa leads to a decrease in the success of reproduction, i.e., a lower number of offspring compared to males with normal sperm [47].

It is worth noting that in the domestic cat, the differences between inbred and outbred litters had disappeared by the time of weaning. It is possible that external factors (for example, food supply ad libitum) neutralized the genetic effect on the survival/body mass of kittens. In the Eurasian lynx, the compensatory growth of small cubs at birth led to the end of lactation and equalization of the body mass of cubs [81], in both inbred and outbred litters [19].

In our study, the most significant predictor for the number of surviving offspring was the degree of parental similarity in the MHC genes. It is believed that choosing a mate with the most distinct MHC genes allows females to produce the most viable offspring. The high polymorphism and polygenicity of MHC molecules, the codominant type of inheritance and the ability of each antigen-presenting cell to express several different MHC molecules make it possible for T-cells to present a wide variety of antigenic peptides. Such a high polymorphism of the MHC genes ensures the body’s resistance to many pathogens and, thus, increases its viability [20,21,22]. It is “beneficial” for parents to bring offspring from a partner that is more distant in terms of MHC genes, providing maximum heterozygosity for the offspring, which increases its viability. It is believed that MHC heterozygotes may have a better life span [82]. In our study, the parents with the maximum distance in the MHC genes I and II classes had more surviving kittens. At the same time, this effect was most pronounced immediately after birth, that is, from parents with a minimum distance in the MHC genes, a significant percentage of kittens were either stillborn or died in the first day after birth. By the age of transition to solid food, this effect was no longer so pronounced. In parents with different distances in class I MHC genes, differences in the number of living offspring at the age of 60 days were also observed, but the effect was already weaker. However, differences in the degree of parental similarity in class II MHC genes in no way affected the number of surviving offspring at the age of 60 days.

It should be borne in mind that, as in the case of inbreeding, low diversity in MHC genes can threaten the viability of the population in the long term by increasing susceptibility to diseases [3,4,5,83]. For example, high MHC diversity in many species is known to be associated with increased survival during disease outbreaks [21,84,85]. It is believed that the social lifestyle of domestic cats contributes to their vulnerability to various diseases [1]. In the breeding season, taking into account the prevailing promiscuous mating system, the parasite press becomes stronger, which is consistent with the immune handicap theory [2]. Accordingly, individuals with low polymorphism in MHC genes may not be resistant to these diseases. At the same time, it has been suggested that adaptation to viral diseases is more important for small feline species (including domestic cats) [86], which is associated with heterozygosity for the MHC II genes. However, the data we have accumulated on the immune status of the domestic cat are not yet sufficient to verify this assumption.

An unexpected result in our study was the absence of the effect of inbreeding and distance in the MHC genes on the body mass of kittens, although many studies have found such a relationship, at least with the degree of parental relationship [11,19]. Moreover, kitten body mass had a weak correlation with the litter size. The domestic cat has a high variability in litter size [87]. It is believed that single kittens or kittens from small litters are usually larger at birth, receive more milk and, therefore, are larger at the time of weaning than twins or kittens from large litters [88,89,90,91,92,93]. In our study, the correlation between litter size and kitten body mass was traced only at birth and only at the tendency level. However, by the period of transition to solid food, the body mass of kittens was largely related to the number of surviving offspring. It is believed that larger cubs usually receive more milk and reach a greater body weight at weaning than their smaller siblings [90,94,95]. Our analysis is insufficient to determine the cause and effect relationship of such an effect. We considered the mass of kittens only in certain segments of their ontogenesis. Without a detailed analysis of the dynamics of body mass throughout from the moment of birth to the transition to solid food, it is impossible to unequivocally say whether larger kittens survived, or kittens were larger, because there were fewer of them in the litter.

The survival rate of offspring in a domestic cat can be surmised to be primarily affected by the degree of similarity/difference in the MHC genes of the parents. This effect manifests itself most significantly at the birth of kittens, while the effect of inbreeding itself was weakly expressed. In our opinion, this is quite expected for a domestic cat. It is the most widespread and most abundant feline species [34,35]. It is characterized by high genetic diversity [36,37]. In this case (if the parents are very different from each other), even full siblings may have higher heterozygosity than in inbred wild feline groups (such as the Far Eastern leopard). Moreover, various studies of the characteristics of animal reproductive strategies show a fairly effective mechanism for reducing inbreeding in the population: mating of a female during the mating season with several partners [67,96,97,98,99,100,101,102,103,104,105]. As a rule, multiple mating of females is associated with an increase in the quality of offspring and their better survival [67,97,100,101,102,103,104]. It is believed that due to multiple mating of female mammals, they have the opportunity to choose the “best genes” (or more “suitable” genes) that can increase the viability and sexual attractiveness of offspring [66,97,100,104,106]. It is assumed that females can influence the competition of male sperm, and the assessment of the quality of partners and their genetic compatibility can occur after mating, in the reproductive organs of the female (“hidden choice of females”) [106]. The free-ranging domestic cat is also characterized by mating with several males during the mating period [42,45,47,107]. Such a mating system in a free-ranging cat leads to an increase in their reproductive success [47] and to the appearance of litters with multiple paternity [42,45,47], although it requires maintaining greater pressure on the immune system [108]. We assume that in such a situation, with a high genetic diversity, the risk of inbreeding in natural conditions is not high. Even when daughters remain in maternal territories and the likelihood of mating with their father during the mating season is high, mating with multiple males can reduce the likelihood of inbred offspring. With the appearance of offspring from an „undesirable” combination of MHC genes, most likely the mortality of these offspring will be quite high and a smaller number of them will survive to reproductive age. These assumptions may hold true for feral felines as well, adjusted for population densities that never approach that of free-ranging domestic cats, and mate selection and mating with multiple mates can be extremely important to the conservation of natural populations. In this case, the negative effects of inbreeding and low MHC variety in free-ranging domestic cats and wild felines are more likely to manifest themselves in populations with a very low density, where the number of partners is limited or in captivity [109]. To manage free-range cat populations, it should be understood that for populations with a high density, the negative effects of genetic similarity of parents are likely to be poorly expressed. In such a situation, it is most likely that no special measures are required. All the negative effects of low genetic diversity will be compensated by a sufficient number of partners, whereas in populations with low density and insufficient food resources, these effects will be more pronounced, and a large percentage of offspring will not live to reproductive age. However, the consequences can be delayed inbreeding and a low diversity in MHC genes. Therefore, in such populations, there may be reproductively active individuals that are weakened and vulnerable to diseases. For such populations, it is necessary to monitor the spread of pathogens and measures to prevent the spread of diseases.

The management of the population of stray cats and dogs is a very difficult task for the human society. Stray domestic cat populations play an important role in urban and rural ecosystems. Their negative effect is expressed mainly by hunting native vertebrate and invertebrate species, and the transfer of pathogens causing zoonotic diseases. On the other hand, domestic cats hunt several rodent species, which may decrease the risk of transfer of other pathogens to humans. The basis for the management of any cat population is the knowledge about the demographic parameters of their population: reproduction rate, reproductive success, mortality rate at different demographic groups and factors affecting these parameters. Thus, our results should be useful in developing a strategy for managing populations of free-range cats. Knowing the impact of low diversity in MHC genes on the survival of offspring will help predict the demographic situation in the population. With a high population density, the number of offspring who have survived to reproductive age can be very high due to the diversity of mating partners and promiscuity. In such populations, the number of animals can increase very quickly. At the same time, such populations can be more resistant to the pressure of parasites and various pathogens due to the high variability in offspring by MHC genes. These data on reproductive success and mortality rate, depending on the diversity of MHC genes (based even on small numbers of loci), can be used for modeling and the prognosis of future population status. Inclusion (animal transfer) or exclusion (sterilization or other methods) of some individuals to/from reproduction may change the basic parameters of population dynamic of stray cat populations. Taking into account that such data may be collected noninvasively (analyzing feces samples), the control of genetic diversity may become a useful instrument in the management of the domestic cat population.

## 5. Conclusions

Thus, the inbreeding coefficient and distance in MHC genes affect reproductive success even in a species with high genetic diversity. Inbred partners had a lower number of kittens alive at birth than outbred ones. However, the most significant predictor for the number of surviving offspring was the degree of parental similarity in MHC genes: the parents with the maximum distance in MHC genes had more alive kittens. Moreover, this effect was most pronounced immediately after birth. A significant percentage of kittens from parents with a minimum distance in MHC genes were either stillborn or died on the first day after birth. By the age of transition to solid food, this effect was no longer so pronounced. Furthermore, neither the inbreeding coefficient nor the distance in MHC genes of parents had any effect on the body mass of kittens. However, we believe that in wild populations, such an effect from the genetic similarity of parents can manifest itself only in populations with a very low density, where the number of partners is limited.

## Figures and Tables

**Table 1:** Effects of IC, litter size and age period on the number of live kittens and average juvenile body weight in domestic cat.

Predictors	Statistics
Number of surviving kittens	
Intercept	B = 0.24 ± 0.38, z = 0.61, *p* = 0.54
Age	B = 0.05 ± 0.3, z = 0.16, *p* = 0.87
IC	B = −1.21 ± 0.73, z = 1.61, *p* = 0.11
Litter size	B = 0.77 ± 0.09, z = 8.12, *p* < 0.0001
Age/Litter size	B = −0.16 ± 0.07, z = 2.19, *p* = 0.03
Body mass of alive kittens at birth	
Intercept	B = 0.12 ± 0.01, z = 22.16, *p* < 0.0001
Litter size	B = −0.004 ± 0.002, z = 1.83, *p* = 0.07
Number of live kittens	B = 0.003 ± 0.002, z = 1.12, *p* = 0.26
Body mass of alive kittens at the 60th day of age	
Intercept	B = 0.88 ± 0.05, z = 16.56, *p* < 0.0001
Litter size	B = −0.02 ± 0.01, z = 1.71, *p* = 0.09
Number of live kittens	B = −0.03 ± 0.02, z = 2.05, *p* = 0.04

B and SE to model-averaged parameter estimates and standard errors in linear mixed-effect models. Litter identity was fitted as a random term in all LMMs. All effects were significant (*p* < 0.05).

**Table 2:** Number of kittens in litters with different litter size and IC.

	Average Litter Size	Number of Kittens Born Alive	Number of Kittens Alive at the 60th Day of Age
Large litters (5 and more)	6.25 ± 0.16	5.0 ± 0.46	4.12 ± 0.58
Small litters (1–2)	1.53 ± 0.13	1.2 ± 0.11	1.2 ± 0.11
Outbreed (0)	3.75 ± 0.24	3.13 ± 0.23	2.95 ± 0.24
Inbreed (0.5)	3.11 ± 0.39	2.11 ± 0.3	2.0 ± 0.36

**Table 3:** Effects of genes of the major histocompatibility complex I (MHC I) and II class (MHC II), litter size and age period on the number of live kittens and average juvenile body weight in domestic cat.

Predictors	Statistics
Number of surviving kittens	
Intercept	B = −0.74 ± 0.95, z = 0.76, *p* = 0.45
Age	B = 0.46 ± 0.36, z = 1.24, *p* = 0.22
MHC I	B = 2.62 ± 0.99, z = 2.55, *p* = 0.01
MHC II	B = −1.41 ± 0.7, z = 1.94, *p* = 0.05
Litter size	B = 0.74 ± 0.12, z = 6.13, *p* < 0.0001
Age/Litter size	B = −0.28 ± 0.09, z = 2.8, *p* = 0.005
Number of alive kittens at birth	
Intercept	B = −0.84 ± 0.64, z = 1.28, *p* = 0.2
Litter size	B = 0.74 ± 0.09, z = 8.16, *p* < 0.0001
MHC I	B = 2.55 ± 0.82, z = 3.0, *p* = 0.003
MHC II	B = −1.23 ± 0.53, z = 2.23, *p* = 0.02
Number of alive kittens at the 60th day of age	
Intercept	B = 0.07 ± 1.45, z = 0.05, *p* = 0.96
Litter size	B = 0.46 ± 0.14, z = 3.14, *p* = 0.002
MHC I	B = 2.52 ± 1.27, z = 1.92, *p* = 0.05
MHC II	B = −1.55 ± 0.99, z = 1.52, *p* = 0.13

B and SE to model-averaged parameter estimates and standard errors in linear mixed-effect models. Litter identity was fitted as a random term in all LMMs. All effects were significant (*p* < 0.05).

## Data Availability

The data presented in this study are available on request from the corresponding author.

## Supplementary Materials

The following are available online at https://www.mdpi.com/article/10.3390/ani12020165/s1, File S1: IC; File S2: MHC.
